# Effect of Al/Ni Ratio on the Microstructure and Properties of Nickel–Aluminum Bronze Alloys

**DOI:** 10.3390/ma17061330

**Published:** 2024-03-14

**Authors:** Yinxun Tan, Haofeng Xie, Xue Feng, Shuhui Huang, Wenjing Zhang, Lijun Peng, Wanyu Wang, Yizhi Zhao

**Affiliations:** 1 State Key Laboratory of Nonferrous Metals and Processes, China GRINM Group Co., Ltd., Beijing 100088, China; 2 GRIMAT Engineering Institute Co., Ltd., Beijing 101407, China; 3 General Research Institute for Nonferrous Metals, Beijing 100088, China

**Keywords:** nickel–aluminum bronze alloys, Al/Ni ratio, mechanical property, corrosion resistance, microstructure

## Abstract

To study the effect of aluminum and nickel elements on the microstructures and properties of the nickel–aluminum bronze (NAB) alloy, four kinds of alloys with different compositions, ZCuAl7–7–4–2, ZCuAl8-6-4-2, ZCuAl9-5-4-2, and ZCuAl10-4-4-2, are prepared by vacuum-melting technology. The effects of different Al/Ni ratios on the microstructures of NAB are investigated using a metalloscope, scanning electron microscopy, transmission electron microscopy, and XPS analysis. The mechanical property is evaluated with microhardness testing and tensile mechanical testing. The corrosion resistance is evaluated using mass-loss testing, electrochemical testing, and corrosion-product characterization. The results show that with the increase of the Al/Ni ratio, the content of precipitated phases increases, while β′ and hard κ, which have a different morphology, appear. As the Al/Ni ratio rises from 1 to 2.5, the hardness increases from 104 HV to 202 HV, and the tensile strength increases by 394 MPa from 356 MPa to 751 MPa, but the elongation decreases substantially from 50.50% to 11.00%. The best corrosion resistance is shown on ZCuAl7-7-4-2, with a corrosion rate of 0.00267 mm/a after 30 d of static immersion corrosion in 3.5 wt.% NaCl solution. Through electrochemical testing and corrosion-product characterization, it is found that ZCuAl7-7-4-2 has the largest polarization resistance R_p_, and the selective corrosion of the surface is mild.

## 1. Introduction

Nickel–aluminum bronze (NAB) alloy is developed based on aluminum bronze by adding Ni, Fe, and other elements to aluminum bronze, eliminating the precipitation of γ_2_ in the casting process. The NAB alloy has good seawater corrosion resistance, anti-cavitation corrosion resistance, and anti-dirt resistance. It also has excellent thermal and electrical conductivity, abrasion resistance, and welding performance. In terms of price, cast NAB is much cheaper than cast super duplex stainless steel or super austenitic stainless steel [1], which has been widely used in structural parts of ships, such as valves, pumps and propellers in the marine environment. The main NAB grade is currently in service for C95800, with 9 wt.% Al, 4 wt.% Ni, 4 wt.% Fe, and 2 wt.% Mn. Its tensile strength R_m_ and yield strength R_p0.2_ can reach 630 MPa and 250 MPa, respectively, and the elongation A is about 16% [2].

Due to the kinds of alloying elements, a series of phase transition reactions occur during the cooling process [3]. It leads to a phase composition of NAB that is relatively complex at room temperature. The typical microstructures include α, β′, as well as κ (κ_I_, κ_II_, κ_III_, and κ_IV_). The α is a matrix phase with good ductility and low hardness. The β′, which has a martensitic structure and is susceptible to corrosion, is a metastable phase retained at room temperature due to incomplete β eutectoid transition at high temperatures [4]. The κ phases are hard and play a role in reinforcement, of which the κ_I_, κ_II_, and κ_IV_ are iron-rich phases (based on Fe_3_Al), but κ_III_ is a NiAl phase (based on NiAl) [5,6,7]. The content and morphology of these phases are greatly affected by the content of alloying elements and the cooling rate during the melting and casting process [8], which play an important role in the differences in mechanical properties and corrosion resistance of NAB.

The Al content greatly affects the morphology of the precipitated phase of NAB [9]. When the Al content increases, the content of high-strength κ_II_ and κ_III_ increases, and the content of high-plasticity matrix α decreases. As the Ni content increases, the solid solubility of Ni atoms in the matrix α increases, which can enhance solid solution strengthening [10]. In the corrosion process, Ni can promote the growth of the surface oxide film and inhibit the formation of corrosion-prone β′, leading to the static corrosion resistance rising and the electrode potential and polarization resistance increasing. With the increase in Fe content, the α is coarsened but still elongated [11]. When Fe content increases, the strength and hardness of NAB increase, while the plasticity is almost unchanged. The addition of Mn can promote the matrix α refinement. Mn is usually added together with Fe. After adding Fe and Mn, Ni enrichment will occur in the precipitation phase and corrosion-product film of NAB [12].

Zhang [13] found that the microstructures and properties of the alloys were investigated when the Al content range varied from 5 to 13 wt.% by modulating the elemental content of Al in aluminum bronzes. It was found that when the Al content was in the range of 5~7 wt.%, the matrix phase was all solid-solution α-Cu and the corrosion was all homogeneous corrosion on the α. At the same time, the thickness of the protective oxide film Al_2_O_3_ formed was thin with poor densification. When the Al content exceeded 9.4 wt.%, the β′ appeared after the alloy was cooled to room temperature. 

Yang [14] studied the microstructures and properties of NAB with different Ni content. She found that the content of the β′ gradually decreased after Ni increased from 4.5 wt.% to 10 wt.%, and the β′ completely disappeared when it reached 8 wt.%. The α is coarsened, and the κ content increases. The yield strength and hardness of the alloy increase by 8.3 and 3.6%, respectively, and the tensile strength and elongation decrease by 15.4 and 55.1% respectively. The increase in corrosion resistance was nearly 50% after 28 d of static immersion corrosion.

J. Basumatary and Wood [15] found that NAB samples immersed in 3.5 wt.% NaCl solution for 3 months formed an oxide film consisting of Al_2_O_3_ and Cu_2_O with a thickness of 1–1.2 μm, which is much thicker than the film-forming thickness of less than 20 nm after oxidation in air. S.S.M. Tavares et al. [16] found the κ_III_ to be lamellar by annealing NAB at 675 °C for 6 h. It was found that the κ_III_ was partially spheroidized, and the β′ disappeared, while the fracture of the tensile sample showed dimples under the scanning electron microscope (SEM) despite the macroscopic brittleness. Shivraman Thapliyal [17] used stir-stirring friction processing to refine the surface grains. The smallest size was only 14.82 μm, which played the role of fine grain strengthening, and the hardness and strength were improved. The microstructure became more homogeneous, while the phenomenon of selected corrosion was reduced, and the corrosion resistance was more excellent.

In summary, many scholars have already investigated the effect of changing a single element or process on the microstructure and properties of NAB. However, few of them have studied the effect on NAB of multiple elements varying together. In this paper, based on the objective of further improving the strength and corrosion resistance of NAB, researching the effect of variation in Al/Ni ratio on NAB under the condition that Fe (4 wt.%) and Mn (2 wt.%) content in NAB remains unchanged. Emphasis is placed on deciphering the relationship between the Al/Ni ratio, microstructure, mechanical properties, and static immersion-corrosion resistance.

## 2. Experimentation

### 2.1. Material Preparation

The compositions of the four NAB alloys are shown in Table 1, and the Al/Ni ratio was increased from 1 to 2.5.

The raw materials were pure copper, pure aluminum, pure nickel, pure iron, and pure ferromanganese (all with 99.99 wt.% purity, Beijing COMPO Advanced Technology Co., Ltd., Beijing, China), which were weighed and cast in ingots according to the design criteria. The weight of each alloy was 4 kg. The materials were dried in a blowing furnace at 60 °C for 2 h before the melting process to ensure that there was no residual moisture on the surface. During the melting and casting process, a medium-frequency vacuum induction furnace (ZG-0.005, Jinzhou Zhongzhen Electric Furnace Co., Ltd., Jinzhou, China) protected by high-purity argon gas (with 99.999% purity, Air Products, Trexlertown, PA, USA) was used for melting. After the completion of the process, the materials were poured into molds, waited for cooling to room temperature, and then were removed from the molds after removing the surface oxidation layer to obtain the as-cast alloys for the experiments.

### 2.2. Experimental Methods

The microstructure of the samples was observed using a metalloscope (OM, Axiovert 200 MAT, Carl Zeiss AG, Oberkochen, Germany), scanning electron microscope (SEM, JSM-F100, JEOL Corp., Akishima, Japan), and Field-emission Transmission Electron Microscope (TEM, TECNAI F20, FEI Company, Waltham, MA, USA). XRD analysis was carried out on oxide films using an X-ray diffraction (Smart Lab, Rigaku Corporation, Tokyo, Japan) with a scanning range of 20° to 100° and a scanning speed of 2°/min. X-ray diffraction phase analysis of the cast NAB alloys was carried out using JSM-F100 (JEOL Corp., Akishima, Japan) diffractometer, with the corrosive agent of 3 g FeCl_3_ + 5 mL HCl + 95 mL CH_3_COOH. Five millimeters of incident light and acceptance slit were selected, and the target material was Cu-Kα. The tube voltage was 40 kV, with a tube current of 30 mA, and the scan speed was 2 °/min. The samples used for TEM were ground to 50–60 µm, then punched into a circle of diameter 3 mm and electrolytically polished with a mixed solution of CH_3_OH:HNO_3_ = 3:1. X-ray photoelectron spectroscopy (XPS) analysis of the corrosion product film was performed using an X-ray photoelectron spectrometer (K-alpha, Thermo Scientific, Waltham, MA, USA) with a monochromatic Al Kα X-ray source (hν = 1486.6 eV) to determine the differences in the composition distribution throughout the film.

For mechanical properties, the WILSON VH1150 Vickers hardness tester (Buehler, Lake-Bluff, IL, USA) was used to measure the hardness of the alloy, and the tensile strength and elongation of the alloy were tested according to the requirements of GB/T 228-2002 “Metallic Materials–Normal Temperature Tensile Test” [18]. In the hardness test, the maximum load was 5 N, and the dwell time was 15 s. Tensile tests of three specimens of each alloy were carried out; afterward, the fracture was observed by scanning electron microscope.

The static immersion-corrosion samples measured 30 × 15 × 2 mm, with a 1 mm circular hole for lifting, and were immersed in a container containing 3.5 wt.% NaCl solution. It was placed in a thermostatic water bath at 25 °C for 30 days, and the solution was changed every 7 days during the test period. After the test was completed, the samples were immersed in a hydrochloric acid solution (concentrated hydrochloric acid mixed with water 1:1) for 3 min while ultrasonic vibration was carried out, and the samples were placed in a drying oven. The mass loss of the samples before and after corrosion was compared to calculate the corrosion rate of the alloy. Parallel sample groups without acid washing were taken, and small pieces of 3 × 15 × 2 mm were cut off. Surface morphology and the oxide film layer after corrosion were observed by scanning electron microscopy.

The electrochemical testing was carried out on three electrode systems using an electrochemical workstation (VersaaSTAT4, Princeton Applied Research, Oak Ridge, TN, USA). The reference electrode required for the tests was an Ag/AgCl electrode. The auxiliary electrode was a platinum electrode. The working electrode was an immersed NAB with a working surface area of 1 cm^2^, and the test solution was 3.5 wt% NaCl solution. Electrochemical impedance spectra were measured after the open circuit was stabilized, and the scanning frequency was in the range of 0.01~10^5^ Hz, with a disturbance signal of 10 mV near the open-circuit potential. In the subsequent determination of dynamic potential polarization curves, the potential scanning was in the range of −0.5 V~+0.8 V, and the scanning rate was 0.5 mV/s.

## 3. Results and Discussion

### 3.1. Microstructure Observation

**Figure 1:**
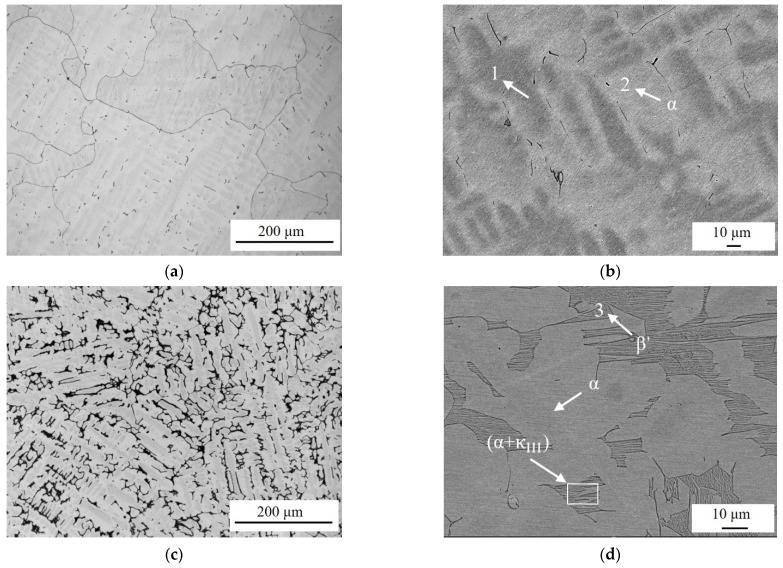

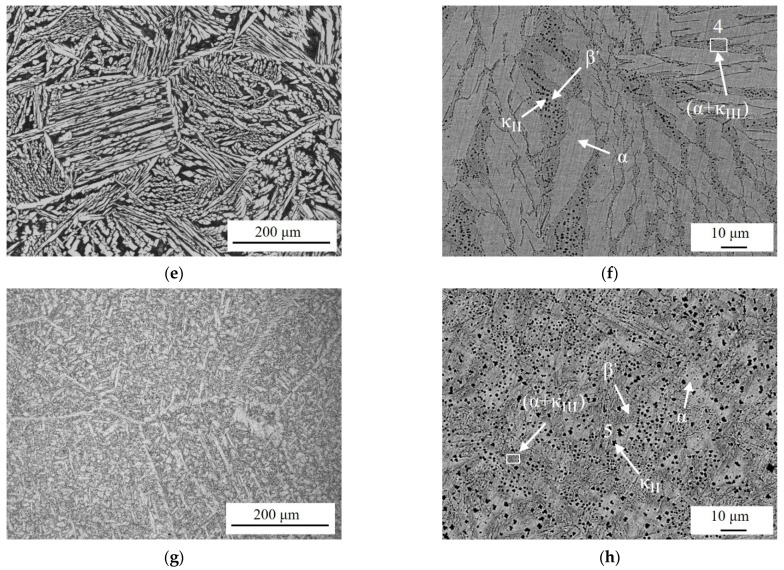
Metalloscope and SEM photographs of NAB alloys: (**a**,**b**) ZCuAl7-7-4-2; (**c**,**d**) ZCuAl8-6-4-2; (**e**,**f**) ZCuAl9-5-4-2; (**g**,**h**) ZCuAl10-4-4-2.

Metalloscope and SEM photographs of NAB with different Al/Ni ratio contents are shown in Figure 1. At room temperature, the typical microstructure of as-cast NAB includes the copper-rich matrix α, the residual martensite β′, and the κ (κ_I_, κ_II_, κ_III_, and κ_IV_) of different compositions and morphologies.

The light grey-scale part of the metallographic photographs shows the α, and the dark grey-scale part shows the β′ and κ. The microstructures of the four alloys are very different, and precipitated phases increase significantly with the improvement of the Al/Ni ratio. In Figure 1a,b, the grain of alloy is an equiaxed grain, with a large grain size when the Al/Ni ratio is 1. ZCuAl7-7-4-2 is dominated by the matrix α, in which dark grey areas are observed under the SEM. A small amount of precipitated phase also exists and is mainly distributed at grain boundaries. In Figure 1c,d, as the Al/Ni ratio is increased to 1.3, the grain still remains in the morphology of the equiaxed grain, but the size of the α becomes smaller. The amount of (α + κ_III_) eutectoid structure increases significantly, forming a continuous lath-like morphology. The increase in the Ni-rich κ_III_ leads to a reduction in Ni segregation in the matrix α. β′ starts to appear in the alloy. In Figure 1e,f, elongated α appears in the alloy at an Al/Ni ratio of 1.8, and the content of β′ increases. At the same time, a large content of spherical or petal-like κ_II_ is distributed between the β′ and (α + κ_III_) eutectoid structure. The slaty (α + κ_III_) eutectoid structure is distributed between the α and β′. In Figure 1g,h, when the Al/Ni ratio increases to 2.5, the α phase decreases dramatically, forming a thin, narrow and short discontinuous morphology, and the proportion of β′ increases significantly. κ_II_ increases in content and becomes larger, and the proportion of κ_II_, which is in the form of petal-like morphology, increases.

Since the Fe in each alloy component does not exceed 5 wt.%, the κ_I_ [19,20] is not detected. The EDS results are shown in Table 2, where it can be seen that there is not much difference in the compositions of the α and β′.

The κ_III_ is a NiAl phase, and the κ_II_ is a Fe-rich phase. The dark grey area in Figure 1e shows elevated Al and Fe elements compared with the matrix α, indicating that this is a microsegregation based on its distribution.

**Figure 2:**
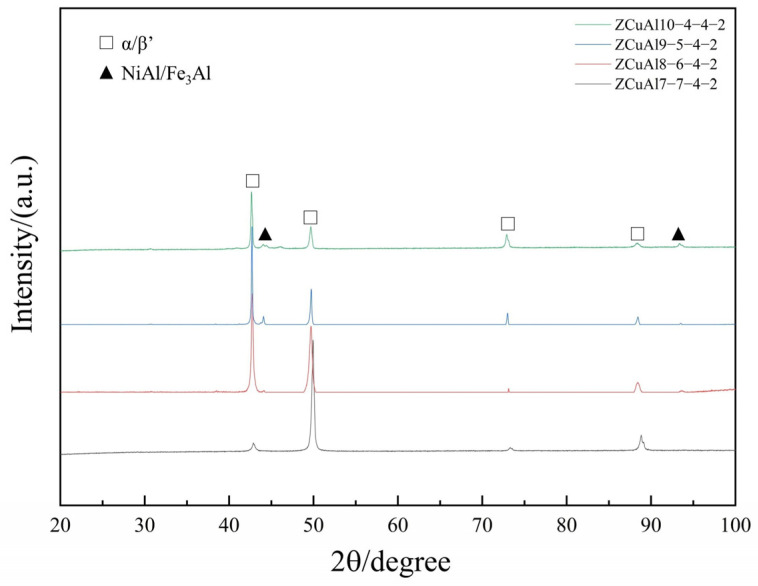
XRD plot of NAB alloys.

The XRD diffractograms of NAB alloys with different Al/Ni ratios are shown in Figure 2.

α, β′, and Fe_3_Al are face-centered cubic, while NiAl is body-centered cubic. The XRD results showed that α and β′ were detected in the alloys, and the peaks of the (Fe, Ni) Al phase were reduced with the decrease in the Al/Ni ratio of the NAB alloys, which confirms that the content of the precipitated phases in the alloys decreases at this time. Due to the large content of solid solution elements in the ZCuAl7-7-4-2 alloy, the intensity of the strongest Cu peak changed from 43° to 50°. At the same time, due to the small content of precipitated phases, no obvious diffraction peaks of the (Fe, Ni) Al phase were found.

**Figure 3:**
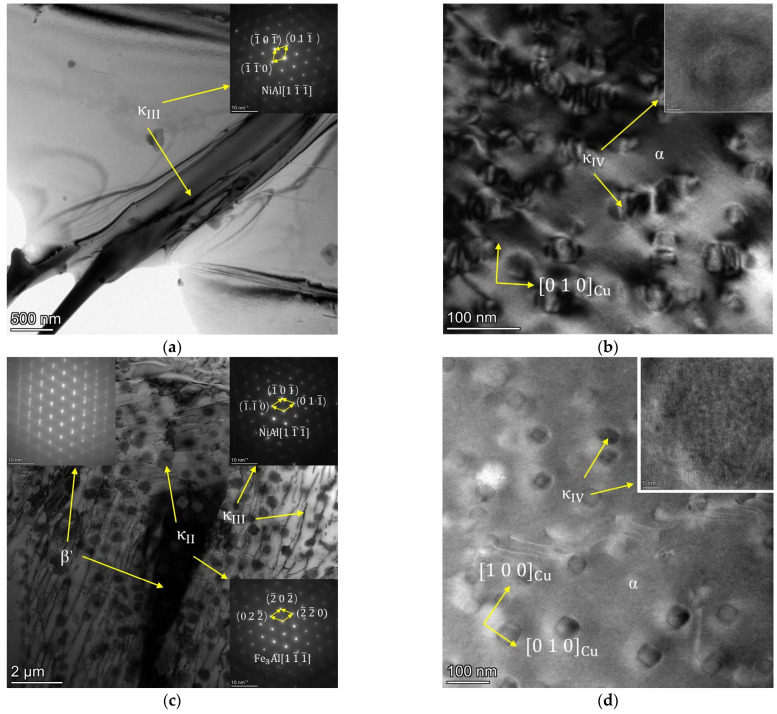
TEM photographs of NAB alloys: (**a**,**b**) ZCuAl7-7-4-2; (**c**,**d**) ZCuAl9-5-4-2.

To further explore the composition of phase in NAB alloys, TEM testing was performed, and the results are shown in Figure 3.

The precipitated phase located at the grain boundaries is observed in the TEM of the ZCuAl7-7-4-2 in Figure 3a, which can be identified as the κ_III_ by analyzing its energy spectrum and lattice. However, due to the low Al content in ZCuAl7-7-4-2, the amount of κ_III_ generated in the eutectoid transformation was small, and thus the formation of continuous lamellar κ_III_ is impossible. By magnifying the picture in Figure 3b, it can be observed that there are many small cubes, with sizes ranging from tens to hundreds of nanometers distributed on the matrix α. The edges of the cubes are parallel to the [1 0 0] and [0 1 0] planes of the substrate Cu and are enriched in Fe, and thus, it can be determined that this is the κ_IV_ that has not been observed previously in the metalloscope or scanning electron microscope. In addition, no significant β′ was observed in ZCuAl7-7-4-2, and the scholars [13,14] found that the NAB with high Ni content and low Al content can effectively inhibit the formation of the β′ during the melting process. In Figure 3c,d, typical precipitation phases of NAB, such as spherical κ_II_, lamellar κ_III_, and fine κ_IV_, among which κ_II_ and κ_IV_ are both dominated by Fe_3_Al, exist in ZCuAl9-5-4-2 alloy. The fact that κ_IV_ is so fine may be related to the reasons for the smaller ingot, higher cooling temperature, and faster cooling rate during the melting and casting process.

### 3.2. Mechanical Testing

**Figure 4:**
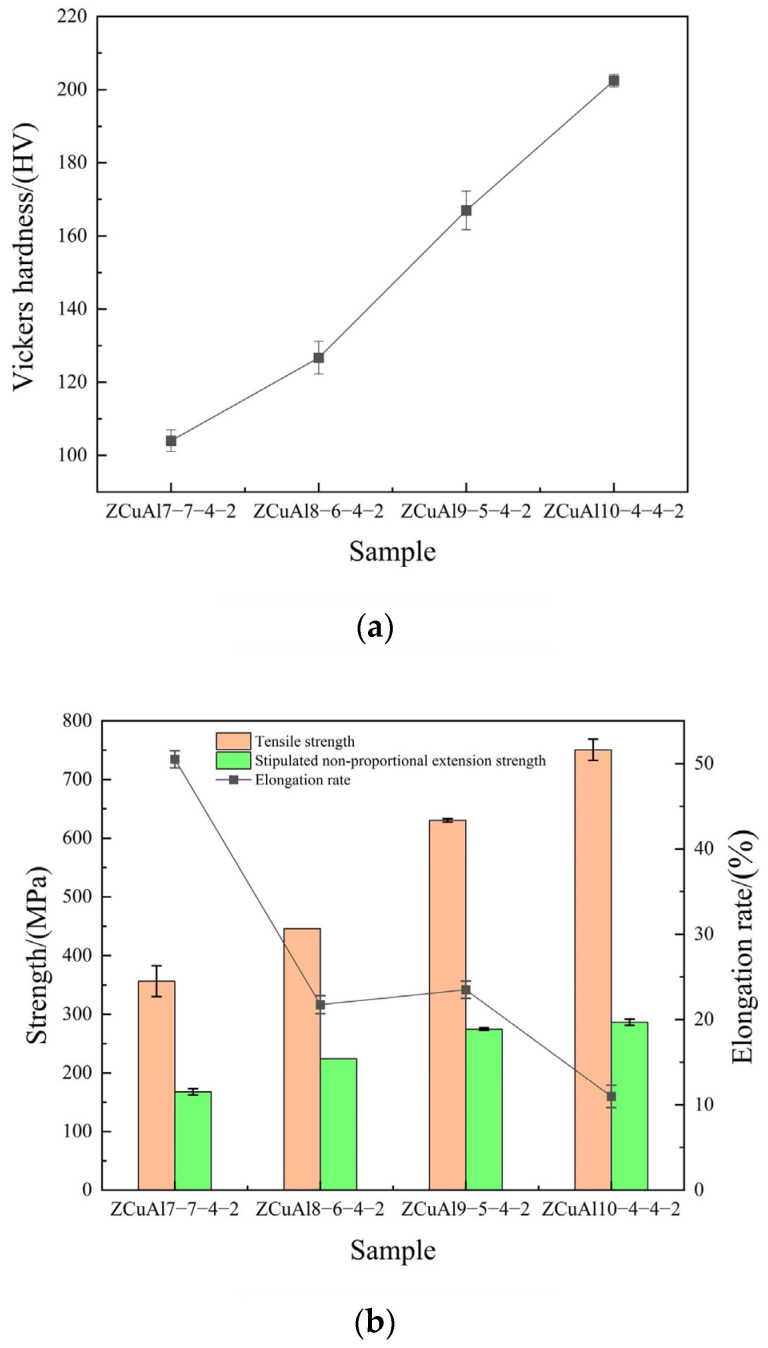
Mechanical properties of NAB alloys: (**a**) hardness and (**b**) strength and elongation.

The mechanical properties of NAB alloys are shown in Figure 4.

As the Al/Ni ratio increases from 1 to 2.5, the content of κ increases significantly, which plays an important role in precipitation strengthening. The hardness and tensile strength of the alloy increase substantially, from 104 HV to 202 HV and 356 MPa to 751 MPa, respectively, by 94.64% and 110.67%, while the plasticity decreases gradually, and the elongation decreases from 50.50% to 11.00%.

**Figure 5:**
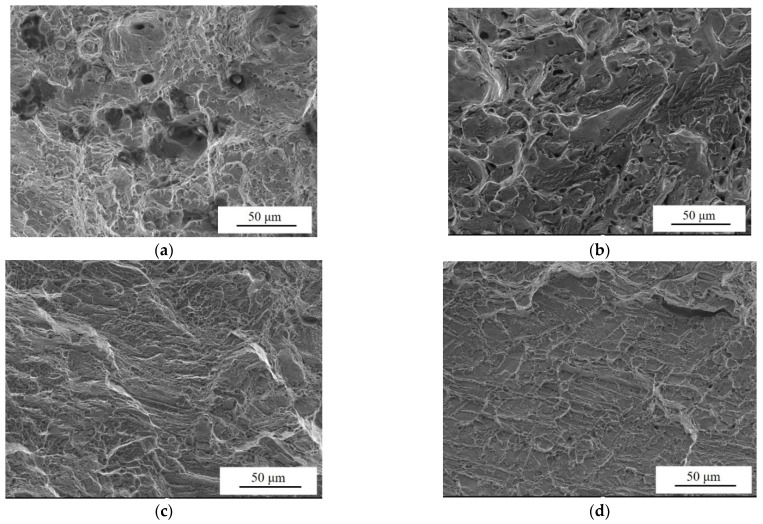
Tensile fracture morphology of NAB alloys: (**a**) ZCuAl7-7-4-2; (**b**) ZCuAl8-6-4-2; (**c**) ZCuAl9-5-4-2; (**d**) ZCuAl10-4-4-2.

The fracture morphology of NAB after stretching is shown in Figure 5.

In Figure 5a, ZCuAl7-7-4-2 has many large-size deep dimples at the fracture, which have obvious ductile fracture characteristics corresponding to its excellent elongation. On the other hand, the content and depth of dimples at the fracture of ZCuAl8-6-4-2 are reduced, and the plasticity of this alloy decreases, as shown in Figure 5b. As the Al/Ni ratio continues to increase, the material gradually evolves toward brittle fracture. The flatter cleavage planes in Figure 5d replace the previous lots of dimples, and the polyhedral morphology of each grain on the tensile fracture resembles the accumulation of icing sugar cubes, which is a typical feature of intergranular brittle fracture [21]. A certain amount of cleavage steps was found in the fracture morphology of NAB with different Al/Ni ratios. This is because when the alloy undergoes deformation, the hard κ phases in the alloy get along with the preferential production of microcracks, which continue to expand and grow and eventually connect and macroscopically cause the alloy to fracture, leaving cleavage steps at the fracture. Therefore, as the amount of the κ precipitation phases in the NAB becomes larger, the content of such cleavage steps also becomes larger.

### 3.3. Static Immersion Corrosion

The annual corrosion rate of the NAB alloy is converted using the Formula (1) [22]:

(1) $$R=\frac{8.76\;\times \;10^{7}\;\times \;M}{\mathrm{STD}}$$

In this formula, R is the corrosion rate (mm/a), M is the mass loss of the sample (g), S is the surface area of the specimen (cm^2^), T is the corrosion time (h), and D is the density of the sample (kg/m^3^).

**Figure 6:**
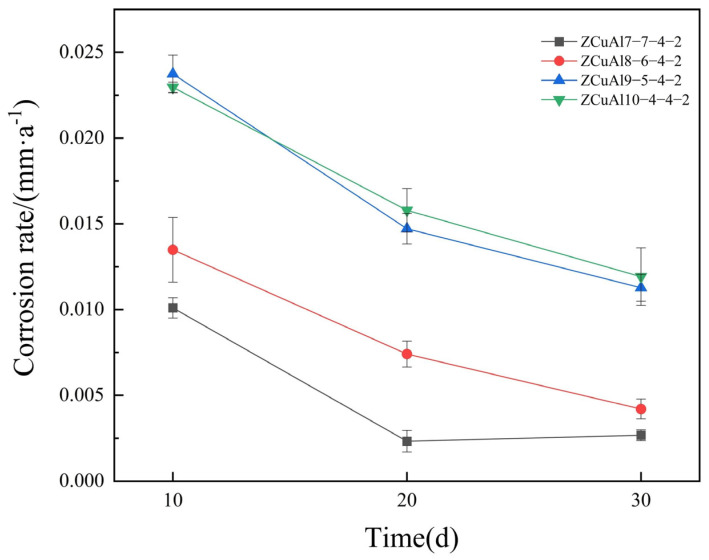
Corrosion rates of NAB alloys after static immersion corrosion for different days.

The corrosion rates of NAB alloys after static immersion corrosion for different days are shown in Figure 6.

Overall, for the same days of immersion in 3.5 wt.% NaCl solution, the corrosion rates of NAB alloys first increase gradually with the increase in Al/Ni ratio and then become close to the same. The corrosion rate of ZCuAl7-7-4-2 is the lowest, while the corrosion rate of ZCuAl8-6-4-2 is slightly higher than the former, and the corrosion rates of ZCuAl9-5-4-2 and ZCuAl10-4-4-2 are close to each other and relatively worse than before. Combined with the microstructure analysis of these NAB alloys, it shows that when there are only fewer precipitated phases, the large and continual Cu-rich α would exist. It results in reduced selective corrosion between the corroded α and κ, and therefore, the corrosion resistance is better. When the proportion of κ in the phase is greater, the Al_2_O_3_ generated after static immersion corrosion on the κ protects the κ from further corrosion, so the corrosion rate does not continue to increase significantly with the increase in the Al/Ni ratio.

With the increase in corrosion time, the corrosion rate of NAB decreased steadily. The ZCuAl7-7-4-2 corrosion rate in 20 d and 30 d was 0.00233~0.00267 mm/a; the corrosion rate tended to be constant. It indicates that at this time, the oxide film was stable, and the exchange of substances between the corrosion solution and the substrate of the alloy was more difficult, while corrosion resistance improved.

### 3.4. Electrochemical Testing

The dynamic potential polarization curves of the oxide film on the surface were tested using the Tafel method. The polarization resistance was calculated according to the Stern–Geary Equation (2):

(2) $$R_{p}=\frac{b_{a}\;\times \;b_{c}}{2.303(b_{c}-b_{a})\;\times \;i_{\mathrm{corr}}}$$

In this formula, b_a_ is the anodic Tafel slope, b_c_ is the cathodic Tafel slope, i_corr_ is the self-corrosion current density, and R_p_ is the polarization resistance.

**Figure 7:**
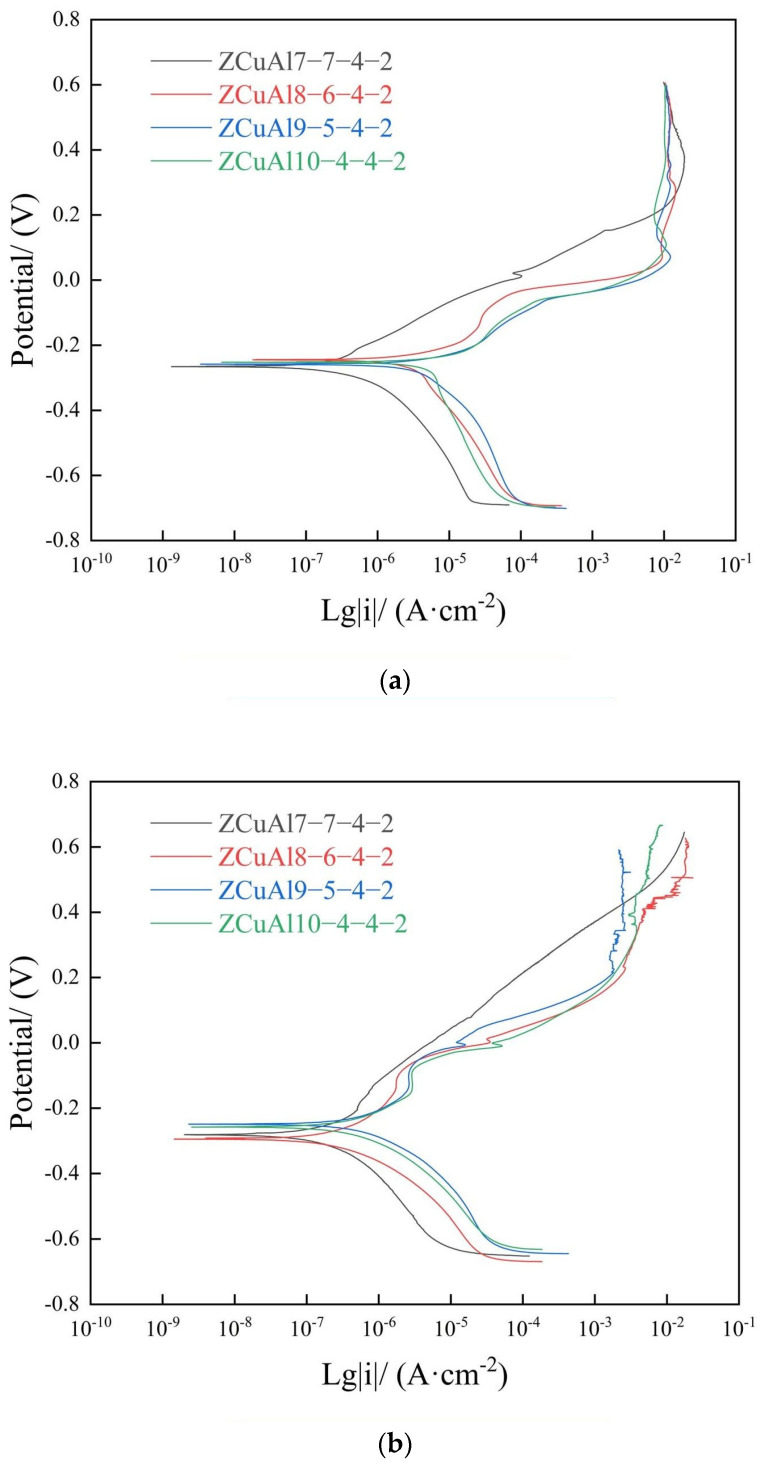
Dynamic polarization curves of NAB alloys after static immersion: (**a**) 10 d; (**b**) 30 d.

Figure 7 shows the dynamic polarization curves of different NAB alloys after static immersion.

In Figure 7a, after immersion for 10 d, pseudo-passivation occurred in ZCuAl7-7-4-2 when the voltage was around 0 V. After that, the i_corr_ continued to increase with the potential to increase, and it started to decrease and stabilize at around 0.4 V, which proves that the oxide film is passivated. Moreover, the i_corr_ of ZCuAl8-6-4-2, ZCuAl9-5-4-2, and ZCuAl10-4-4-2 started to go to a steady state at 0.2 V.

In Figure 7b, after 30 d of static immersion, the anodic polarization curves of ZCuAl8-6-4-2, ZCuAl9-5-4-2, and ZCuAl10-4-4-2 showed obvious passivation in the dynamic polarization curves, with an increase in voltage up to about 0.2 V, indicating that the dense oxide film was generated on the surface [23]. After that, the potential continued to increase, and i_corr_ tended to stabilize. The fitted data are shown in Table 3. In the case of corrosion for the same days, ZCuAl7-7-4-2, with the lowest Al-Ni ratio, always has the smallest self-corrosion current i_corr_ and the largest polarisation resistance R_p_.

**Figure 8:**
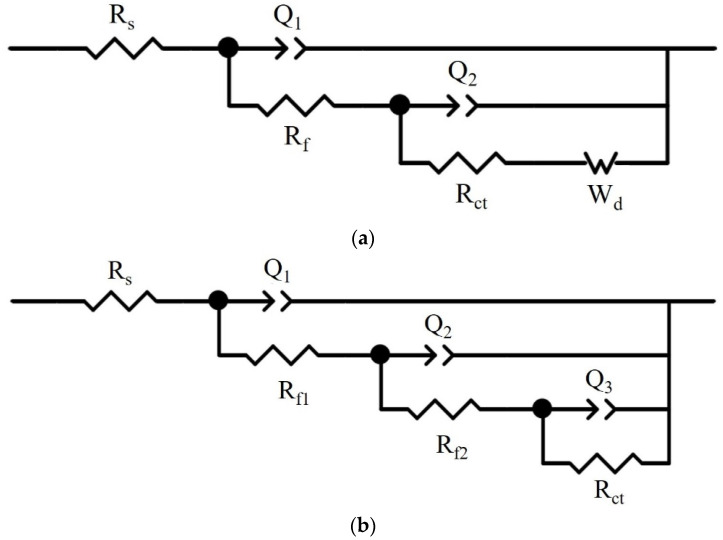
Fitting equivalent circuits: (**a**) R(Q(R(Q(RW)))); (**b**) R(Q(R(Q(R(QR))))).

The electrochemical impedance spectra (EIS) were carried out on the surface of NAB, and the equivalent circuit [24] of Figure 8 was used to fit the results. It is observed that there are three peaks in the Bode plot of ZCuAl7-7-4-2, proving that there are three time constants.

Therefore, the equivalent circuit in Figure 8b was used to fit ZCuAl7-7-4-2, and the equivalent circuit in Figure 8a was used for the remaining three alloys, which is a commonly used one in the description of NAB alloy corrosion. R_s_ is the solution resistance; R_f_ is the film resistance and R_f_ = R_f1_ + R_f2_; R_ct_ is the charge transfer resistance; the polarization resistance R_p_ can be expressed as R_p_ = R_f_ + R_ct_; Q is a constant phase element (CPE); W_d_ is the Warburg impedance.

**Figure 9:**
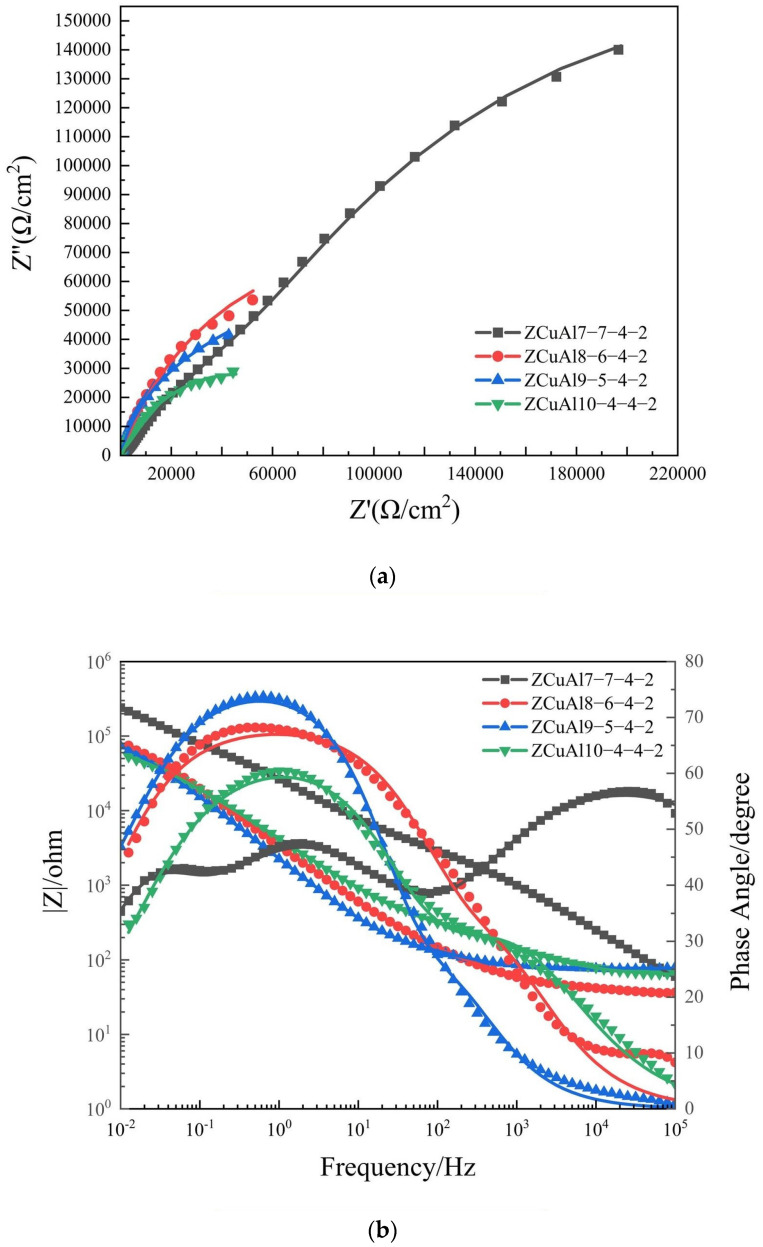
30d EIS of NAB alloys: (**a**) Nyquist plot; (**b**) Bode plot.

The resulting plots are shown in Figure 9, respectively, with the symbols and lines representing test data and fitted data.

The Nyquist plots are mainly composed of high-frequency capacitive arcs representing the charge-transfer resistance and the surface-film properties [25,26]. The Warburg impedance [27], which represents the diffusion of O_2_ from solution across the oxide film to the electrode surface or the diffusion of ${\mathrm{CuCl}}_{2}^{-}$ from the electrode surface across the oxide film, is very small. It proves that after 30 d of corrosion in 3.5 wt.% NaCl solution, a stable oxide film was generated on the surface, and it is difficult for O_2_ and Cl^−^ in the corrosion solution to pass through the oxide film and react with the alloy substrate; thus, the corrosion rate of NAB is mainly affected by the charge transfer step. The radius of the capacitive arc of the ZCuAl7-7-4-2 with the lowest Al-Ni ratio is greater than that of others, so it has the largest R_p_ theoretically.

In the Bode plot, the |Z| values decreased with the increase of the Al/Ni ratio in the low-frequency region after a static immersion of 30 d. This indicates that ZCuAl7-7-4-2 has the largest R_p_, and the corrosion resistance of NAB degrades theoretically. In the phase angle diagram, ZCuAl8-6-4-2 had the widest peak width in the mid-frequency region of the phase angle, indicating that the oxide film was the thickest among these NAB alloys. The low-frequency region of ZCuAl7-7-4-2 consisted of two smaller peaks, and the highest peaks were in the high-frequency region, while the highest peaks of the remaining three alloys were all in the low-frequency region. Table 4 shows the fitted data of EIS, and ZCuAl7-7-4-2 has the highest R_p_ with excellent corrosion resistance, which has the lowest Al–Ni ratio.

### 3.5. Corrosion Products and Oxide Films

**Figure 10:**
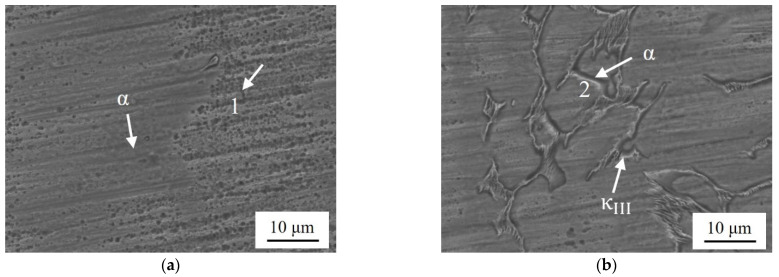

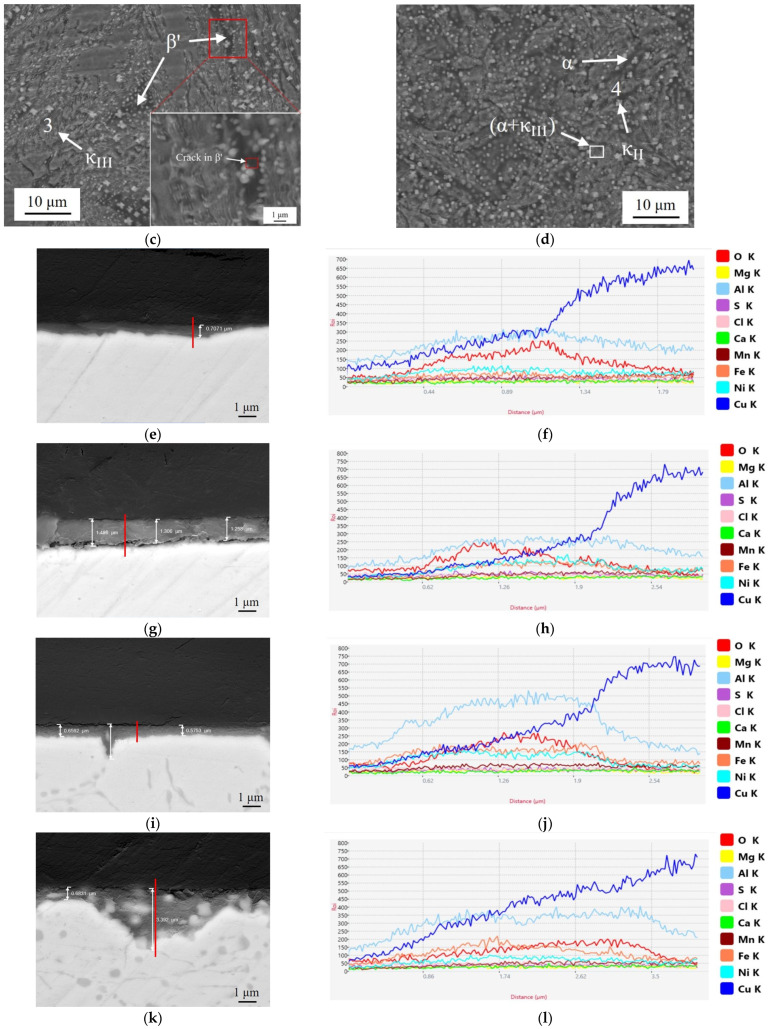
Surface morphology, oxide film morphology, and EDS line scanning plots of NAB alloys after static immersion for 30 d: (**a**,**e**,**f**) ZCuAl7-7-4-2; (**b**,**g**,**h**) ZCuAl8-6-4-2; (**c**,**i**,**j**) ZCuAl9-5-4-2; (**d**,**k**,**l**) ZCuAl10-4-4-2.

Figure 10a–d shows the surface corrosion morphology of NAB after 30 d of static immersion. The surface shows different degrees of corrosion.

In Figure 10a, the phase of ZCuAl7-7-4-2 is mainly matrix α, with a diffuse distribution of many small κ_IV_ precipitation phases in the matrix. In areas where microsegregation was previously present, the surface is corroded more severely relative to the matrix α, as indicated by Label 1. With the rise of the Al/Ni ratio, there are precipitated κ_II_ and κ_III_, and these phases will rapidly generate Al_2_O_3_ oxide film in a neutral Cl^−^ solution, which makes the corrosion potential higher than matrix α and β′. This results in the β′, which is near these κ phases that are corroded first, followed by α, with a clear selective corrosion phenomenon [28]. As shown in Figure 10b–d, the highlighted white part of electron microscopy is the κ that has generated a protective oxide film, and the surrounding deep black is the corroded α and the β′. The more the content and density of precipitated phases, the more obvious this phenomenon of phase-selective corrosion is, and thus, it can correspond to the results of static immersion corrosion. Overall, ZCuAl7-7-4-2 has the most uniform surface after being corroded.

Figure 10e,g,i,k shows the morphology of the oxide film of NAB after 30d of static immersion. With the increase in the Al/Ni ratio, observations on the oxide film of NAB alloys revealed that the oxide film thickness first increases and then decreases. Among them, the film layer of ZCuAl8-6-4-2 was the thickest, up to 1.2–1.5 μm. The maximum corrosion depth gradually increased, and the maximum corrosion depth of ZCuAl10-4-4-2 reached more than 3 μm, while the inside of the oxide film was attached with white snowflake-like corrosion products. The maximum corrosion depth appeared near the position of β′ and κ_III_ on the surface, which is characteristic of selective corrosion. The results of EDS line scanning of the oxide film with the red line position in the figure are shown in Figure 10f,h,j,l. Compared with the substrate composition, Cu can be seen in the oxide films rising with the increase of the depth of the oxide film. Al, Ni, and Fe show obvious enrichment phenomena, and the degree of enrichment rises with the increase of the Al/Ni ratio. The content of O in the film exceeds the substrate by a lot, showing the phenomenon of increasing first and then decreasing, basically disappearing to the position of the substrate, which corresponds to the results of the EDS test in Table 5.

Mn does not have an obvious enrichment phenomenon. Therefore, in summary, the corrosion resistance of NAB decreases with the increase in the Al/Ni ratio.

According to the known literature [29], in a neutral Cl^−^ solution, the corrosion process of NAB is as shown in Equations (3)–(7). In the first step, Cu is the anode corrosive dissolution, and oxygen undergoes oxidation at the cathode. The reaction equation is the following:

(3) $$\mathrm{Cu}+2{\mathrm{Cl}}^{-}-e^{-}\to {\mathrm{CuCl}}_{2}^{-}$$

(4) $$O_{2}+2H_{2}O+4e^{-}\to 4{\mathrm{OH}}^{-}$$

(5) $$2{\mathrm{CuCl}}_{2}^{-}+2{\mathrm{OH}}^{-}\to {\mathrm{Cu}}_{2}O+H_{2}O+4{\mathrm{Cl}}^{-}$$

During this process, a protective film of Cu_2_O is formed. In addition, the Al undergoes the following chemical reaction during the corrosion process, producing Al_2_O_3_ [30]:

(6) $$\mathrm{Al}+4{\mathrm{Cl}}^{-}\to {\mathrm{AlCl}}_{4}^{-}+3e^{-}$$

(7) $${2\mathrm{AlCl}}_{4}^{-}+3H_{2}O\to {\mathrm{Al}}_{2}O_{3}+6H^{+}+8{\mathrm{Cl}}^{-}$$

Cu_2_O can also be further oxidized to produce loose Cu_2_(OH)_3_Cl [31], with Cl^−^ aggregation in the film. This also proves that the enrichment of Al, Ni, and Fe in the oxide film is because the main components of the oxide film are Cu_2_O, Al_2_O_3_, and nickel–iron oxides, which can play a protective role.

**Figure 11:**
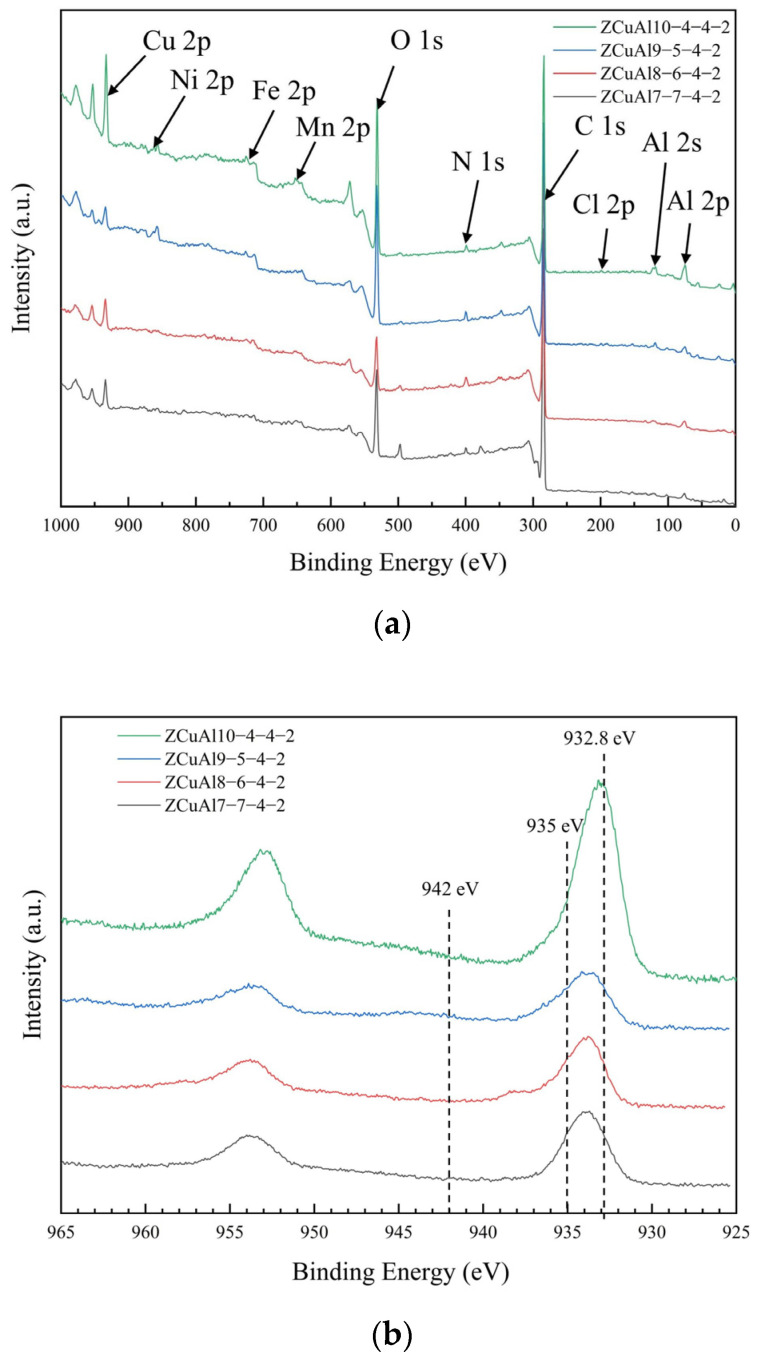

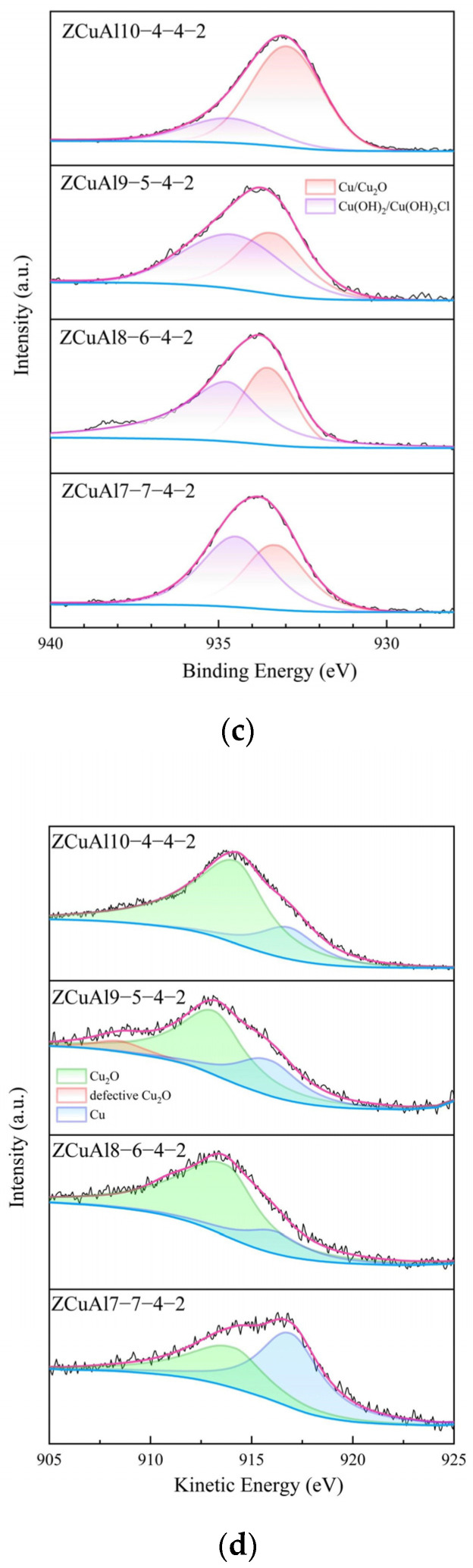
XPS testing results: (**a**) XPS full spectrum; (**b**) Cu 2p mapping; (**c**) fraction of Cu 2p mapping; (**d**) Cu LMM mapping.

To further investigate the composition of the oxide films of NAB after static immersion for 30 d, XPS analysis is carried out on the surface, and the results are shown in Figure 11.

Figure 11a shows that the oxide film is mainly composed of Cu, Al, Ni, Fe, Mn, and O, of which the O peak is very high, indicating that there is a large content of oxides on the surface at this time. There is little Cl from the corrosive medium, which is consistent with the SEM energy spectrum results, indicating that the content of copper chloride hydroxides is scarce.

In Figure 11b, the presence of a strong peak at a binding energy of ~932.8 eV is evidence of the presence of Cu or Cu_2_O [32,33]. The combination of a weak peak at ~935 eV and a significant oscillation at ~942 eV suggests that an amount of Cu(OH)_2_ or Cu_2_(OH)_3_Cl may be present [34], which may be due to the oxidation of Cu_2_O during storage or testing. It is also found that there is a shift in the peak located at ~932.8 eV for all alloys, except for ZCuAl10-4-4-2, which is less pronounced. A fit to this fraction of Cu 2p is shown in Figure 11c. This indicates that these three alloys have a higher Cu(OH)_2_ or Cu_2_(OH)_3_Cl content on the surface of the oxide film than ZCuAl10-4-4-2 in the XPS analysis region.

Since the binding energies of copper (~932.8 eV) and cuprous ions (~932.7 eV) in Cu 2p are close to each other, it is difficult to differentiate between them. Therefore, the distinction was made in the luminescence multiplexing microscopy (LMM) of Cu, and the results are shown in Figure 11d. The percentage of Cu_2_O in the oxide film increases with the increase in the Al/Ni ratio.

**Figure 12:**
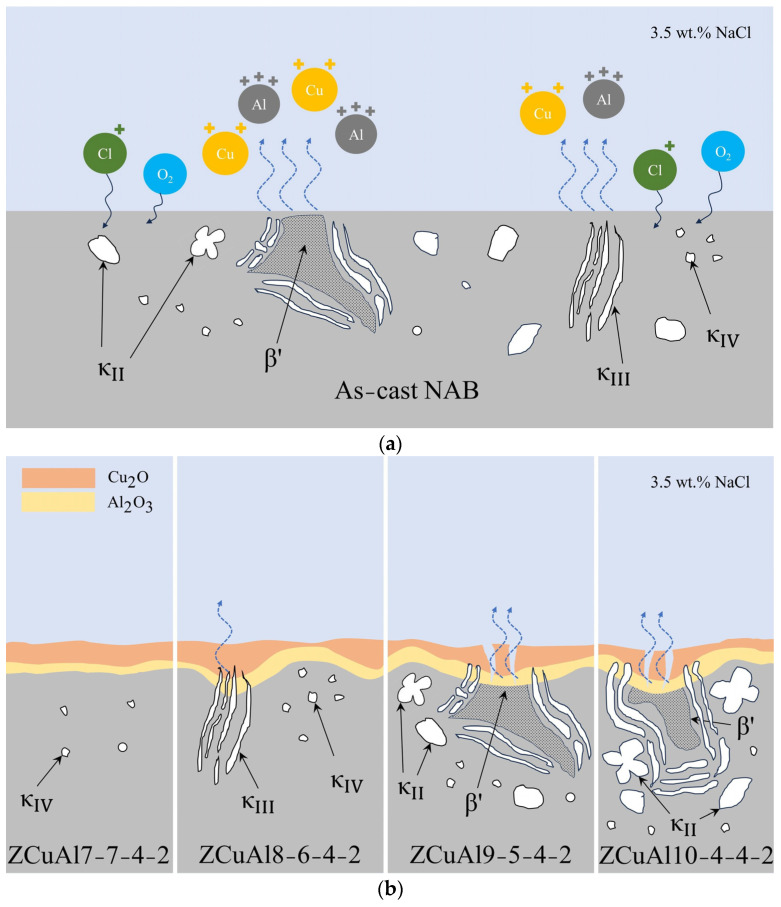
Schematic diagram of selective corrosion mechanism for NAB: (**a**) NAB before corrosion; (**b**) four kinds of NAB alloys with different Al-Ni ratios after corrosion.

Combining the above observations and studies on the corroded surface and oxide film, the corrosion process model, as shown in Figure 12, can be established.

At the early stage of corrosion of NAB in 3.5 wt.% NaCl solution, the κ has the lowest potential due to the rich Fe_3_Al and a high content of Al and is preferentially corroded to form Al_2_O_3_ on the surface, which becomes the inner layer of the final oxide film. After that, Al_2_O_3_ covering the κ makes the potential increase. After that, Cl^−^ starts to corrode the β′, which has a martensitic structure and unstable chemical state firstly, and then corrodes the matrix α close to the κ to form ${\mathrm{CuCl}}_{2}^{-}$, which eventually hydrolyzes to Cu_2_O and becomes the outer layer of the final oxide film. In addition, there is a difference in the rate and thickness of corrosion products generated on the surface of the β′ and the surrounding α, which leads to cracks on the surface of β′. It makes the oxide film lose its protective properties, so the corrosion rate of the β′ is faster.

## 4. Conclusions

In this paper, four kinds of NAB alloys with different Al-Ni ratios were prepared with vacuum melting. The mechanical properties were characterized by determining the hardness, strength, and elongation. The static corrosion resistance and process were investigated by using static immersion corrosion and electrochemical workstations. The following conclusions were obtained:

1. The morphology and amount of precipitated phase of cast NAB are greatly affected by the Al/Ni ratio in the composition. As the Al/Ni ratio rises from 1 to 2.5, the β′ begins to appear and increases, and the lamellar κ_III_ and globular, petal-like κ_II_ precipitate sequentially, with an increase in the content of precipitated phases.
2. The Al/Ni ratio can be increased to improve the hardness and strength of the NAB, respectively, from 104.02 HV to 202.46 HV and 356.33 MPa to 750.67 MPa, with an increase of 94.64% and 110.67%. However, the elongation decreases from 50.50% to 11.00%, and the fracture mode of NAB shifts from ductile fracture to brittle fracture, with plasticity deteriorating.
3. Under static immersion corrosion in 3.5 wt.% NaCl solution for the same days, the corrosion rates first increase gradually and then approach unanimity, with an increase in the Al/Ni ratio. With a longer corrosion time, the corrosion rate of NAB decreases steadily. Through the dynamic potential polarization curves testing and EIS, it can be seen that with the increase of the Al/Ni ratio, the self-corrosion current density increases while the oxide film resistance R_p_ decreases. The |Z| value also decreases, which indicates that the corrosion resistance deteriorates.
4. In the corrosion process of neutral NaCl solution, NAB has an obvious phenomenon of selective corrosion. Cl^−^ in the neutral solution first reacts with the Al in the κ, which has a lower potential. It eventually hydrolyzes to form dense Al_2_O_3_ attached to the surface of the κ, which protects the κ from further corrosion, leading to an increase in the potential of the κ at this time. Subsequently, Cl^−^ reacts with β′, which has the lowest potential now, and then reacts with matrix α. It eventually hydrolyzes to form the protective Cu_2_O. The final oxide film thus appears as Al_2_O_3_ on the inner layer and Cu_2_O on the outer layer. Through XPS, it can be found that with the increase of the Al/Ni ratio, the phenomenon of selective corrosion becomes more and more serious, and the content of Cu^+^ in the oxide film gradually increases.

## Figures and Tables

**Table 1:** Measured compositions of NAB alloys.

Alloy	Al(wt.%)	Ni(wt.%)	Fe(wt.%)	Mn(wt.%)	Cu(wt.%)	Al/Ni Ratio
ZCuAl7-7-4-2	6.94	6.82	4.10	1.72	Bal.	1.0
ZCuAl8-6-4-2	7.90	6.13	3.92	2.12	Bal.	1.3
ZCuAl9-5-4-2	8.94	5.18	4.12	2.18	Bal.	1.8
ZCuAl10-4-4-2	9.94	3.99	3.94	1.82	Bal.	2.5

**Table 2:** Results of EDS analyses of different crystalline phases.

Phase	Al(wt.%)	Ni(wt.%)	Fe(wt.%)	Mn(wt.%)	Cu(wt.%)
1	8.88	7.81	5.46	1.79	76.06
2(α)	6.09	7.66	4.09	2.06	80.10
3(β′)	9.51	6.40	2.97	2.26	78.86
4($\kappa_{III}$)	13.15	11.62	9.96	2.44	62.83
5($\kappa_{II}$)	16.11	9.51	44.02	3.65	26.71

**Table 3:** Fitted data of dynamic polarisation curves.

Sample	Exposure Time (D)	b_a_ (mV/Dec)	b_c_ (mV/Dec)	*E*_corr_ (mV)	*i*_corr_ (μA/cm^2^)	*R*_p_ (×10^3^Ω∙cm^2^)
ZCuAl7-7-4-2	10D	199.19	−165.66	−265.72	0.50	78.12
ZCuAl8-6-4-2	10D	77.53	−182.92	−245.06	1.34	17.68
ZCuAl9-5-4-2	10D	72.74	−157.85	−258.23	1.77	12.20
ZCuAl10-4-4-2	10D	73.25	−220.34	−252.73	2.16	11.07
ZCuAl7-7-4-2	30D	327.22	−244.19	−281.48	0.35	173.48
ZCuAl8-6-4-2	30D	201.92	−140.96	−295.55	0.36	100.12
ZCuAl9-5-4-2	30D	205.54	−147.79	−249.35	0.69	54.41
ZCuAl10-4-4-2	30D	211.85	−179.19	−257.75	0.78	54.04

**Table 4:** Thirty-day EIS fitted data for NAB alloys.

	ZCuAl7-7-4-2	ZCuAl8-6-4-2	ZCuAl9-5-4-2	ZCuAl10-4-4-2
*R*_s_/(Ω cm^2^)	5.4	37.3	77.3	60.8
*Q*_1_/(μF cm^−2^ s^n−1^)	2.6	38.5	47.6	33.0
*n*_1_	0.662	0.750	0.820	0.639
*R*_f_ (*R*_f1_)/(Ω cm^2^)	3967	134	144	407
*Q*_2_/(μF cm^−2^ s^n−1^)	8.9	32.9	45.9	31.5
*n*_2_	0.625	0.784	0.877	0.770
*R*_f2_/(Ω cm^2^)	153,900	-	-	-
*Q*_3_/(μF cm^−2^ s^n−1^)	19.2	-	-	-
*n*_3_	0.847	-	-	-
*R*_ct_/(Ω cm^2^)	363,500	196,300	111,400	90,300
*R*_p_/(Ω cm^2^)	521,367	196,434	111,544	90,707
*W*_d_/(×10^−3^ S cm^−2^ s^1/2^)	-	-	0.12	1.76
χ^2^/(×10^−3^)	0.13	3.11	0.58	0.68

**Table 5:** EDS analysis results of different phases after static immersion for 30 d.

Area	Al(wt.%)	Ni(wt.%)	Fe(wt.%)	Mn(wt.%)	O(wt.%)	Cl(wt.%)	Cu(wt.%)
1	6.08	10.42	8.44	1.66	6.92	0.15	66.33
2(α)	7.95	6.07	6.07	2.54	13.87	0.35	63.14
3($\kappa_{III}$)	19.31	18.87	17.23	3.17	13.09	0.42	27.92
4($\kappa_{II}$)	16.88	10.83	24.95	3.42	18.67	1.02	24.24

## Data Availability

Data are contained within the article.
